# Environmental values and attitudes among farmers in China – a case study in the watershed of Yuqiao reservoir of Tianjin Municipality, China

**DOI:** 10.1080/00207233.2016.1220699

**Published:** 2016-08-19

**Authors:** Geir Inge Orderud, Rolf D. Vogt

**Affiliations:** ^a^ NIBR–Institute for Urban and Regional Research, Oslo and Akershus University College of Applied Sciences, PO Box 4 St. Olavs Plass, N-0130Oslo, Norway; ^b^ Department of Chemistry, University of Oslo (UiO), P.O. Box 1033 Blindern, N-0315Oslo, Norway

**Keywords:** Values, Attitudes, Policies, Eutrophication, China, Farmers

## Abstract

Failure to curb water pollution in China brings to the fore the issue of environmental values and attitudes among Chinese farmers. Applying the New Ecological Paradigm Scale this study finds that the pro-environmental value of *New Ecological Paradigm (NEP) Worldview* has a stronger standing among the studied Chinese farmers than the *Dominant Social Paradigm (DSP) Worldview*.

## 1. Introduction

The state of the Chinese environment has in recent decades been painted in bleak colours and assessed to be at the brink of collapse [1,2]. Serious environmental challenges and degradation are confirmed in annual reports from the Ministry of Environment [3,4].^1^ True, pursuing economic growth at the expense of the environment became particularly evident under Premiere Deng in the 1980s and 90s, expressed by his ‘getting rich is glorious’ slogan. Gradually, though, environmental protection made its way up the policy priority ladder; illustrated by a 15% average growth rate in the Chinese environmental protection sector [5] and a number of laws and regulations. Nevertheless, the priority of economic growth is still strong, and decision makers at the local level face the hard task of determining trade-offs between economic growth and curbing environmental pollution.

In the view of Harris [6,7], environmental values generally compete with other priorities, with mixed results regarding giving priority to protecting the environment. Reviewing the literature, Harris concludes that ‘a *policy for the environment* …. requires a change in thinking and a change in attitudes. *It requires environmental values at the heart of environmental policy*’ [6]. On the other hand, Kolmuss and Agyeman [8] underline that studies have failed to link environmental values (and knowledge) to environmental actions. The same conclusion was drawn in a study of actions, using the same sample as this study [9], although we contend that environmental values and attitudes are important elements of policies.

A number of studies have analysed environmental values and attitudes in China (see Section 2), but not many on farmers. Most studies compare broad categories of urban and rural residents. Our study fills a void in the literature by focusing on environmental values and attitudes of residents living in farming villages. Moreover, in China practically all freshwater lakes and water reservoirs are in the process of eutrophication [10]. Leaching of nutrients from farming is the main reason for substandard water quality. The success of abatement actions will thus depend on commitments by farmers. This study concerns the following questions:

(i) What fundamental environmental values are characteristic for Chinese farmers in governing their interaction with nature, and how do these values differ between categories and according to features of farmers?
(ii) How are fundamental environmental values forming and interacting with environment related attitudes?
(iii) How can the revealed environmental values and attitudes be embedded and used in a wider Chinese context of environmental policies? How might a local context frame environmental values and attitudes?

Section 2 below puts the analysis in the theoretical frames of Environmental Behaviour with a focus on environmental values and attitudes, and then goes on to present the quantitative methodology and survey used in the analysis. In Section 3, the first subsection constructs a number of composite variables on the basis of factor analyses of value and attitude questions in the survey. These variables are then analysed by the method of multivariate regression in the second subsection. Findings produced by the empirical analyses are discussed in Section 4, and main conclusions presented in Section 5.

## 2. Theory, methodology, and empirical basis

### 2.1. The theoretical frame

This study belongs to the field of Environmental Behaviour, and applies the New Ecological Paradigm (NEP) scale [11]. The Environmental Behaviour theory applied here has environmental values as one pillar alongside situational and psychological factors [12].

The NEP scale is claimed to represent ‘beliefs about nature and humans’ role in it’ as the statements^2^ ‘constitute a fundamental component of people’s belief systems vis-à-vis the environment’ [11]. Moreover, it has been argued [13] that the NEP scale is also theoretically related to a basic cultural value of harmony–mastery, and that this is a basic aspect of human society–nature interaction. These interactions may lead to harmonious relations as represented by *New Ecological Paradigm* (*NEP*), or to exploitation as represented by a *Dominant Social Paradigm* (*DSP*). The 15 statements consist of 8 statements representing a *NEP worldview* and 7 statements represent a *DSP worldview*; see Annex 2 for a listing of statements, with the *NEP* and *DSP* statements listed with odd and even numbers, respectively.

Hawcroft and Milfont [13], in a review of studies applying the NEP scale, criticised many studies for ignoring statistical constraints, possibly causing flawed results (e.g. the impact of sample type on score profile and the use of varying number of statements). Moreover, testing the number of dimensions being measured by the NEP scale, a study [14] concluded ‘that these facets are better represented statistically as correlated rather than orthogonal structures’. The empirical analysis of the NEP scale, presented in Section 3, is informed by this literature.

The NEP scale was developed through trials and analyses of results from responses among students in the US. Although not designed to be US specific, the NEP scale may not relate well to Chinese conditions. A study [15] conducted on a Turkish sample takes a critical stance, concluding that the validity of some of the NEP statements did not find empirical support and should be ‘reconsidered, revised or eliminated’. But that Turkish study did not reject the NEP scale as such. Critical studies are also found in China [16–18], but other studies support the use of the NEP scale [19, 20]. We take the same position as Vikan et al. [21] that the ‘amorphousness of the scale …. may actually be a strength because it may show how cultures are similar and different as regards one of humanity’s common problems’.

Some studies of Chinese citizens claim that pro-environmental values are stronger in urban areas and especially in larger cities [19, 20], but another study [22] concluded that ‘residents of Shaanxi province overall appear to have a more instrumental than eco-centric view of the ideal relationship of humans with nature’. Another study [23] concludes that the people in China have a ‘coherent sense of generalized environmental concern’.

Three additional sets of questions regarding the related environmental attitudes were queried in this study (see Annex 2 for a listing of all questions and response options). The first set concerns different categories of environmental challenges and pollution problems: water scarcity and droughts; heatwaves, erosion of fertile soil; loss of biodiversity; particular animal species becoming extinct; polluted drinking water; air pollution; and noise from transportation and industrial production. The second set of questions concerns environmental features that might be taken into account when considering industrial activities in natural environments: presence of endangered animal species; how untouched by human beings the biotope is; the economic value of biotopes and species for human industrial production; and the scenery of a particular landscape and its economic value for tourism. The third set of questions concerns farmers’ motivations for doing farming: achieving high productivity and high output; earning as high an income as possible; producing healthy livestock; achieving environmentally sound production; and being recognised by other farmers for good farming.

The study thus covers both value and attitude variables. Generically, we consider *values* as governing attitudes; the abstract concept of what is right; whereas *attitudes* are tendencies for responding positively or negatively. This means that values of *NEP and DSP worldviews* govern the attitudes of the three other set of questions of our analysis. In reality, *values* and *attitudes* can contradict each other, and feedbacks from practising *attitudes* will influence governing *values*.

Nevertheless, we expect that a prevailing *NEP worldview* would make the person more conscientious about pollution and environmental issues; thereby facilitating motives for achieving environmentally sound farming. Farmers with a *DSP worldview,* on the other hand, should respond in an opposite manner, although they might still be concerned about certain types of pollution (e.g. pollution directly affecting human beings) and certain consequences of activities in natural environments (e.g. having negative consequences for other economic activities). A production oriented farming attitude might be shared by both a *NEP and a DSP worldview*, although stronger by farmers with a *DSP worldview*.

### 2.2. Background and independent variables assessed in the analysis

The analysis includes an assessment of the governing role of traditional background variables like gender, age, education, and income, although their impacts on the environmental values and attitudes are ambiguous (Annex 1). For *gender*, some studies claim females to be more supportive of the environment than males (i.e. more ‘caring’) [24, 25]. Others report no clear impact [26]. Likewise for China, some studies indicate that females are more pro-environmental than males [27, 28], while a cross-national study [29] found that although females were more pro-environmental than men in the private sphere, this difference more or less disappeared in the public sphere. For *age*, it is more about cohort effects, or experiences during formative years [30] than age as such, although it is reported [6] that the young in China are more pro-environmental.

Regarding *education* there is research claiming the highly educated to be most pro-environmental [6, 31], also in China [23]. On the other hand, academic discipline was found to be more important than level of education in influencing the holders environmental values [27, 32], implying that e.g. a short environmental-oriented education combined with work experience might foster stronger pro-environmental values than a long economics-oriented education. Regarding *income*, the environmental Kutznets curve hypothesis [33] and the post-materialist hypothesis [34] claim increasing income and affluence to facilitate a stronger priority of non-polluting production. The rationale is that it becomes affordable to take environmental actions, thereby also facilitating the development of environmental values. On the other hand, villagers in the Chinese countryside have for centuries been practising recycling and reuse of their modest resources. Increasing their income might therefore, at least in the short term, lead to less recycling and reuse with a less frugal lifestyle.

The analysis comprises four additional background (independent) variables: The *village poverty level* was calculated, according to the OECD poverty measure, on the basis of income for all respondents, thereby introducing an *affluence*–*poverty* variable representing the overall economic condition of each village. Whether or not the interviewees had *jobs outside of agriculture* was recorded as a potential proxy for influences from urban life. *Membership of Communist Party of China (CPC)* was recorded as a measure of the respondents’ position in the local community, and resembling priorities within the party. It has been found [35] that CPC membership caused lower feeling of injustice and that existing inequalities were considered less harmful, but Party members also expected that the government would level out any inequalities. Moreover, CPC members are reported to be more concerned about environmental conditions than others [23]. Finally, though most importantly, the questionnaire requested the entrants to state their s*elf*-*reported social status in the village*, reflecting the interviewees’ self-esteem. This is relevant because people claiming to have high social status are assumed to be held in high esteem by their fellow villagers, thereby acting like role models.

**Figure 1.**
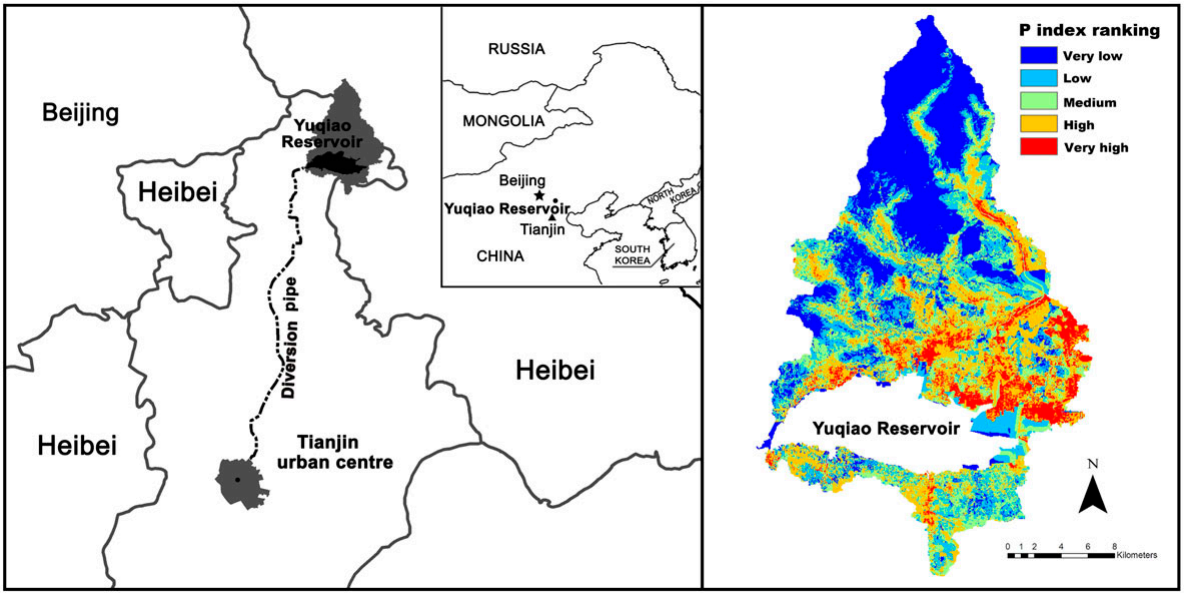
The study area, and the P index rating of the soil; modified from Zhou et al. [36].

### 2.3. Material, empirical basis and methods

The survey, taking place in spring 2012, was conducted in a local watershed situated north of the Yuqiao reservoir, providing Tianjin municipality with drinking water^3^. The study area has more than 100,000 residents distributed in 150 villages, with populations ranging from a few hundred to about two thousand residents. Moreover, there are more than 100,000 pigs, almost a million poultry, about 6,000 cattle and 16,000 sheep [37]. The reservoir is man-made and regularly receives water from the neighbouring Hebei province through a diversion canal. In short, this is a region with a pervasive human imprint.

Farmers in the area apply a mix of manure, human sewage, and mineral fertilisers to increase their produce. Effluent manure and sewage is disposed of on wasteland or directly into drainage channels because there is no centrally organised collection system [38]. Soil in the lowlands mainly consists of shallow layers of tilled soil with poor nutrient absorption capacity above an impermeable layer of clay rich soil^4^ [39]. During heavy rainfall the clay layer constrains vertical percolation. This forces the water instead to flush rapidly through the upper tilled layer, carrying a high load of nutrients into waterways which eventually end up in the reservoir [40]. The right section of figure 1 presents a Phosphorus Index identifying areas with [a] high risk of providing phosphorus to the reservoir.

Leaching of phosphorous from the local watershed, especially during the monsoon rainfall, is causing annual algae bloom, and the reservoir cannot meet the required water standard [36]. The water quality of Yuqiao reservoir has deteriorated in recent decades. Total phosphorous concentration reached an annual value of 50 μg P/L in 2012 [41]. This is higher than the OECD tolerance limit of 30 μg P/L, above which eutrophication is likely to be encountered. Environmental authorities have taken such actions as decommissioning fishponds, establishing constructed wetlands, introducing restrictions on access to the lake, and displacing farmers. The farmers are thus expected to be aware of the negative consequences of their farming practices on the water quality through observing the implementation of the abatement actions, as well as many reporting bad smell and taste of water, or even becoming sick from drinking water^5^.

Residents in 11 villages participated in the survey. The villages were chosen to cover two perpendicular transects spanning the shore of the reservoir and through the lowland plain and up along the main valley. In addition, the selection of villages was based on dominant crops (wheat, corn, vegetables and orchards) and husbandries (pig farming and presence of fish farming). In total, 545 respondents participated, ranging from 39 to 60 respondents in each village, with an average of about 50. Four graduate students conducted the practical tasks of the survey, supervised by the responsible researcher. The students responded to inquiries from respondents, ensuring that the questions were understood, as well as monitoring farmers filling in the questionnaires. This improved the reliability of the data.

The sample is not considered representative for all background variables, especially regarding gender. Achieving something approaching a representative sample would have required following a top-down approach, with County level and Township/Village level government officials conducting the survey. A bottom up approach was instead pursued as this allowed the students to be present so that it was possible to provide assistance to the respondents, Although there is a dominance of women, still about a quarter of the cohort are men; allowing for including gender as an independent variable in the multivariate analyses. The sample had a fairly good distribution on age and education categories.^6^

## 3. The empirical assessment

### 3.1. Basic statistical analysis

Tables in Annex 2 present basic statistical measures (means, standard deviations) for the replies to the four sets of questions being analysed, as well as item-to-total correlations for all the individual statements. Fairly high correlation coefficient with the average of the other statements indicates an acceptable to good reliability of the data. The four sets of questions are also tested by Cronbach Alfa scores, which were found to be in the range of 0.866 to 0.914. This is considered to be excellent, indicating good internal consistency and reliability of the data.

### 3.2. Factor analyses and the making of composite variables

Factor analyses have been conducted by applying the oblimin rotation method, thereby allowing for correlations between statements [14]. Three *statistically based* ‘*factor models’* were produced for each of the four sets of questions (see the tables A–D in Annex 2).

#### 3.2.1. Composite value variables

Based on the factor analyses and the models of each analysis, the following two composite value variables were formulated.

The *NEP worldview* and the *DSP worldview*:

(i) The *NEP worldview* (statements 5, 11, 13, and 15): ‘Humans are severely abusing the environment’; ‘earth is like a spaceship with very limited room and resources’; ‘balance of nature is very delicate and easily upset’; and ‘if things continue on their present course, we will soon experience a major ecological catastrophe’.
(ii) The *DSP worldview* (statements 4, 8, 10, and 12): ‘Human ingenuity will ensure we do not make the earth unliveable’; ‘balance of nature is strong enough to cope with the impact of modern industrial nations’; ‘the so-called ‘ecological crisis’ facing humankind has been greatly exaggerated’; and ‘humans were meant to rule over the rest of nature’.

Comparing the three factor models, it is notable that Model 3 combines the presumed DSP statement of ‘Humans have the right to modify the natural environment to suit their needs’ with several NEP statements, indicating farmers being fully aware of and accepting that this will cause ‘severely abusing the environment’ and ‘disastrous consequences’. Their rationale seems to be that the problem is not serious enough to make the earth uninhabitable and that human ingenuity eventually will fix the problems. There is a strong correlation between the *DSP* statement 6 ‘The earth has plenty of natural resources if we just learn how to develop them’ and the *NEP* statement 7 ‘Plants and animals have as much right as humans to exist’. A possible explanation is that some farmers think that the Earth has plenty of resources for both human beings and animals.

The mean scores for the two new variables (i & ii) indicate that the *NEP worldview* (3.7) is stronger than the *DSP worldview* (3.2). The share with a mean above 4.0 for the *NEP worldview* is 42.6%, and the share with a mean above 4.5 is 17.9%. It is reasonable to consider this latter group of farmers as *potentially* being guided by a strictly environmental perspective and none other; a pro-environmental mind-set. The larger group of about 43% might be interpreted as taking an *environmentalist* perspective; that is, supporting environmental conservation and potentially becoming an environmental activist. Corresponding shares for the *DSP worldview* are 19.2 and 8.6%. Although some farmers are found in the highest category for both worldviews, *farmers’ support for the NEP worldview is considerably stronger than for the DSP worldview*.

#### 3.2.2. Composite attitude variables

##### Pollution and environmental challenges

The three water related issues are used as the basis for one variable in the multivariate regression analysis (Section 3.3), taking into account the central issue of Yuqiao reservoir as a raw water source for drinking water, and high factor scores for the water related issues of the set of questions, as well as high absolute mean score for the water related issues. The three biodiversity related issues of the second factor model are used as the basis for a second variable in the multivariate regression analysis:

(i) Water-related issues: ‘Water scarcity and droughts’; ‘polluted drinking water’; and ‘waste water polluting rivers and lakes’.
(ii) Biodiversity-related issues: ‘Loss of biodiversity’; ‘poaching’; and ‘particular animal species becoming extinct’.

##### Basis for environmental concerns over human expansion into natural areas

Having a *DSP worldview* does not necessarily imply that one is opposed to taking any sort of measures for conserving or protecting certain environmental features that might be threatened by human activities, but that the rationale and reasoning (values) for environmental protection might differ from those of a *NEP worldview*. From factor models 2 and 3, two variables are thus constructed, representing a bio-centric perspective and anthropocentric perspective, respectively:

(i) Bio-centric: ‘Endangered animal species’ and ‘endangered plant species’.
(ii) Anthropocentric: ‘Cultural and historical value of a landscape, biotopes and particular species for human society’; and ‘the scenery of a particular landscape and its economic value for tourism’.

##### Motives for farming

From the three factor models three variables for different motives for farming were constructed:

(i) Production and income: ‘Achieve high productivity and high output’; ‘achieve high quality on the food products’; and ‘earn a high income as possible’.
(ii) Recognition/status: ‘Achieve model farmer status’; ‘recognized by others for good farming’; and ‘recognizes by authorities for good farming’.
(iii) Environment and health: ‘Have healthy animals’; ‘have an environmental sound production’; and ‘produce food for own production’.

**Figure 2.**
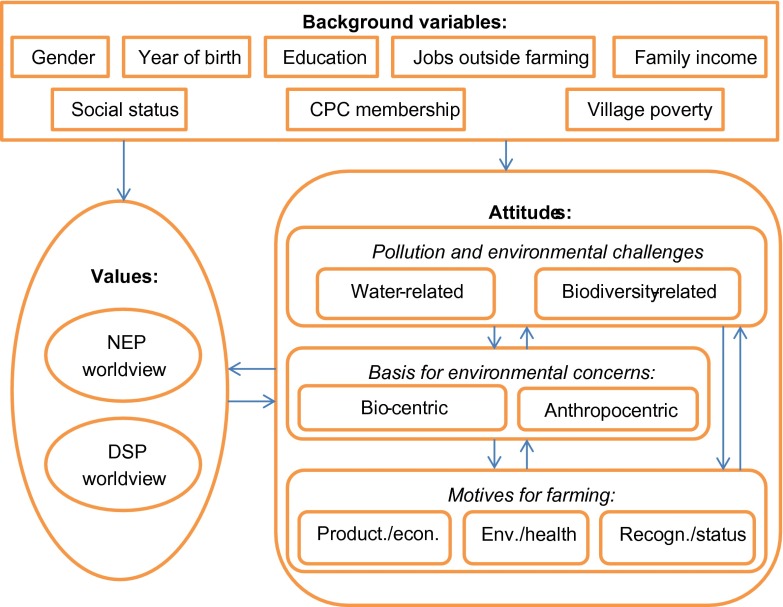
The conceptual linkages between different sets of variables.

Figure 2 below summarises the different sets of variables representing values, attitudes, and structural (background) factors. The idea of two-way linkages is depicted with arrows in both directions between value and attitude variables.

### 3.3. Multivariate regression analyses

The replies to questions are designed as ordinal Likert scales and the (in)dependent variables, constructed on the basis of the factor analyses (Section 3.2), are numerical means. Empirical relationships between the question scores and the combined values and attitude variables are thus possible to study by linear regression models. The method used is hierarchical regression with two blocks of variables, shown as Models in the tables 1 and 2: First, a block (Model 1) of traditional background variables (gender, year of birth, own education, jobs outside farming, family income, self-reported social status in village, CPC membership, and village poverty rate), and then a block of value and attitude variables are added under Model 2 (the NEP scale, pollution issues, environmental features, and farm motives). Variables being significant (*p* < 0.05) in Model 1 might not be so in Model 2. Regression coefficients presented in brackets under Model 1 and Model 2 do not meet the 0.05 criterion, though remain below 0.1.

**Table 1.** Multivariate analysis (linear regression model) of environmental values (NEP scale) and attitudes (environmental features) among farmers.

	NEP worldview		DSP worldview		Environ features: bio-centric		Environ features: anthropocentric	
	Model 1	Model 2	Model 1	Model 2	Model 1	Model 2	Model 1	Model 2
Gender	−.137*							
Year of birth			−.171*	−.153*				
Own education					.145*	.123*		
Jobs outside farming							.146*	.142*
Family income								
Social status village								
CPC membership					.207***	.188***	(.114)	
Village poverty								
NEP worldview						.188***		.138*
DSP worldview								
Pollution: water		.170***						.223***
Pollution: biodivers.				.162***		.278***		
Env.: bio-centric		.212***						
Env.: anthropocentric				.194***				
Motive: prod/econ						.161***		.148*
Motive: env/health		.151***						
Motive: recog/status				.181***				
R^2^	.052	.166	.031	.139	.097	.263	.048	.158
Durbin-Watson		1.916		1.935		1.754		1.864

**Table 2.** Multivariate analysis (linear regression model) of attitudes (pollution and farming motives) among farmers.

	Env/pollution: water		Env/pollution: biodiversity		Motive: prod/econ		Motive: envir/health		Motive: status/recogn	
	Model1	Model 2	Model 1	Model 2	Model 1	Model 2	Model 1	Model 2	Model 1	Model 2
Gender					−.138*	(−.109)				
Year of birth									−.174*	−.146*
Own education		−.128*								
Jobs outside farming										
Family income										
Social status village					.150*	.140*			(.113)	(.111)
CPC membership	.135*									
Village poverty										
NEP worldview		.157***				.143*		.173***		
DSP worldview				.126*						.176***
Pollution: water										
Pollution: biodivers.										
Env.: bio-centric		.234***		.312***		.173***				
Env.: anthropocentric										
Motive: prod/econ										
Motive: env/health										
Motive: recog/status										
R^2^	.049	.140	.027	.144	.065	.124	.031	.059	.085	.115
Durbin-Watson		1.803		1.780		1.770		1.917		1.902

Statistical tests (bivariate correlations, heteroscedasticity with histograms/plots of residuals, collinearity with tolerance/VIF, Durbin-Watson coefficients, and condition indexes) show that the regression analyses are reliable.

#### 3.3.1. The NEP and DSP worldviews analysis

Table 1 shows the correlation coefficients (r) for the regressions between explanatory factors (background variables, values and attitudes) and the two models for each of the worldviews. Model 1 shows that gender is an important explanatory background variable: females are more supportive of the *NEP worldview* than males, but gender disappears in Model 2, when the following three attitude variables are supporting a *NEP worldview*: (i) The bio-centric variable when considering industrial activities in nature; (ii) the water related issue variable under pollution and environmental challenges; and (iii) the environment/health variable under farming motives.

Turning to the *DSP worldview*, Model 1 identifies that the support increases with age. Model 2 reveals that the following attitude variables support the *DSP* worldview: (i) The anthropocentric variable when considering industrial activities in nature; (ii) the recognition/status variable under farming motives; and (iii) biodiversity related issues under pollution and environmental challenges.

Comparing the two worldview values, these results show that variables under the three sets of ‘attitude questions’ generally explain the *NEP worldview* or the *DSP worldview* as expected. The role played by gender and age is also as anticipated, adhering to the previously described perception of the role of these background variables.

#### 3.3.2. Governing environmental factors when introducing industrial activities outside built areas

Table 1 shows the correlation coefficients (r) between the two combined variables reflecting the basis for environmental concern and the other explanatory factors. Model 1 for the *bio*-*centric variable* shows the strongest support by ‘CPC membership’ as well as those with ‘more education’. Both of these variables also show up in Model 2, together with (i) the biodiversity variable under environmental challenge/pollution’, (ii) ‘having a *NEP worldview*’, and (iii) the production/economic variable under farming motives.

Model 1 for the *anthropocentric variable* identifies ‘jobs outside farming’ as supportive. This variable is also present in Model 2, but then together with (i) the water related issues variable under pollution and environmental challenges’, (ii) ‘having a *DSP* worldview’, and (iii) the production/economic variable under farming motives.

Linkages with the values reflected in *NEP*- and *DSP worldviews* are as expected, whereas the linkages with the attitude question variables are unclear, as is also the case for background variables (table 1).

#### 3.3.3. Pollution and Environmental concerns

Table 2 shows the correlation coefficients (r) for the regressions between the two combined attitude variables for pollution and environmental concerns and other explanatory factors. Regarding concern over *water related issues*, Model 1 reveals CPC membership as supportive, but this variable disappears in the case of Model 2, where (i) bio-centric variable when considering industrial activities in nature; (ii) a *NEP worldview*; and (iii) ‘low education’ appear as supportive.

Regarding the *biodiversity related issues* variable, Model 1 does not identify any significant values and attitudes, whereas Model 2 identifies fairly strong support by (i) the bio-centric variable when considering industrial activities in nature; and then also support by (ii) *DSP worldview*.

The linkages with the values reflected in *NEP* and *DSP worldviews* are as expected, whereas the linkages with attitude question variables are mixed, which is also the case for background variables.

#### 3.3.4. Farm motives

Table 2 shows the correlation coefficients (r) between the two combined variables reflecting the three main motives for farming and the other explanatory factors. Some caution is required with this variable since the heteroscedasticity test revealed outliers for all three variables. Yet it should be noticed that (i) increasing social status supports the *production/economic variable*, and that (ii) increasing age supports the *recognition/status* variable, and that these links are showing up for both models. Further, (iii) a *NEP worldview* supports the *production/economic variable* and the *environmental/health variable*, whereas (iv) a *DSP worldview* supports the *recognition/status variable*.

Again, the linkages with the *NEP* and the *DSP worldview* are as expected, and also the link between the social status and age background variables are as expected for one of the variables.

## 4. Discussion

**Figure 3.**
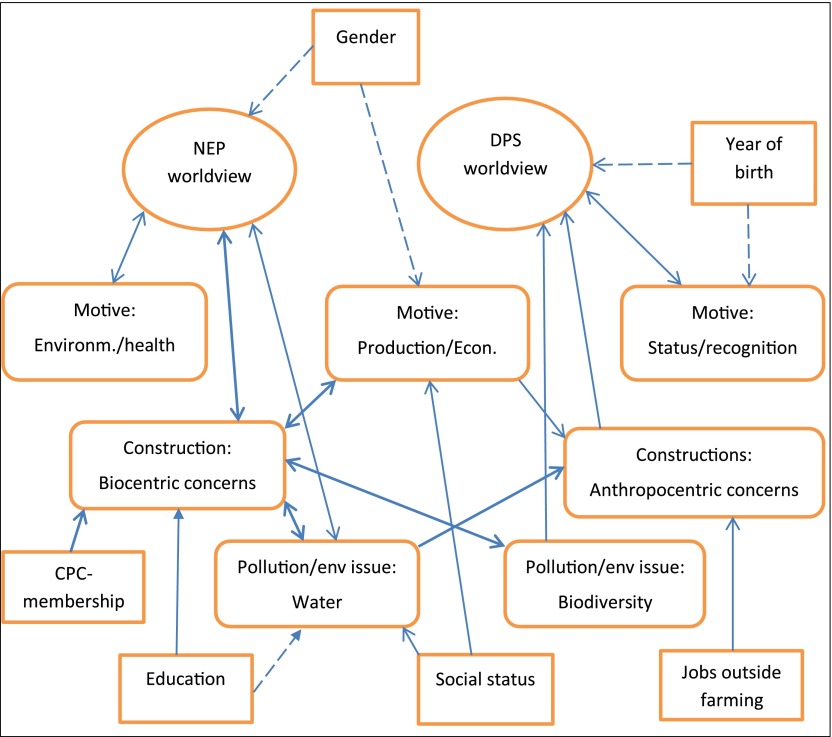
Summary of explanatory linkages between value variables, attitude variables, and background variables.

Notes: The two value variables are in circular boxes, attitude variables in muted squared boxes, and background variables in squared boxes. Positive linkages are presented by full arrow lines and negative linkages with broken lines. Two-way linkages are depicted with arrow in both ends, and linkages with regression coefficients above 0.200 are shown in bold arrow lines.

*Values* are ideas and beliefs that are considered fundamental and *attitudes* are tendencies for responding positively or negatively to e.g. ideas. In Section 2.2 we underlined that the link between values and attitudes is not any simple cause–effect relation but rather a complex interaction including feedback loops. The statistical analysis of the data confirms this complexity. This is also apparent from figure 3 which summarises results presented in tables 1 and 2.

### 4.1. The environmental farmer and the human-centred farmer

The factor analysis (Section 3.2.1) illustrated that farmers based on their values in general may be grouped into either a *NEP worldview* or a *DSP worldview*, with the means indicating a stronger support for the *NEP worldview*, thereby countering previous studies arguing this to be generally an urban phenomenon. In addition there is a third and smaller segment which is factoring on one of the *DSP* statement and some *NEP* statements. Similarly, the factor analyses of the attitude questions reveal that farmers on certain dimensions are grouping into segments that can be characterised as either pro-environmental or human-centred, but the linkages are complex and tend to blur the lines between different positions regarding the environment.

Differences within *NEP worldview* are mainly explained by endangered species (bio-centric), environment/health farming motivation, and water issue variables, whereas differences within the *DSP worldview* are mainly explained by anthropocentric, recognition/status, and biodiversity related issues. Generally, these linkages show that pro-environmental attitudes are governed by pro-environmental values of a *NEP worldview*. Correspondingly, human-centred attitudes are governed by the values of a *DSP worldview*. But we also see that the biodiversity related variable is linked to the DSP worldview, indicating some mixing of pro-environmental and human-centred values and attitudes.

Reversing the linkages makes the pattern more blurred: (i) For the issue of taking into account environmental factors when planning and implementing industrial activities in natural environments, having a *NEP worldview* means higher scores on both the bio-centric variable and the anthropocentric variable than having a *DSP worldview*; (ii) for the issue of pollution and environmental challenges, having a *DSP worldview* is explanatory for the biodiversity related issues variable; and (iii) for farming motives, the *NEP worldview* is linked to both the production/income variable and environment/health variable. This entails that the *NEP worldview* is the strongest predictor for the anthropocentric variable, and the *DSP worldview* is the strongest predictor for the pro-environmental biodiversity related issues variable. The *NEP worldview* is also the strongest predictor for the production/income variable in spite of this variable having a strong human-centred element, but also possibly a pro-environmental element. This reflects and illustrates well the dire trade-off challenge confronted by the farmers.

Taking into account the linkages between attitude variables makes the picture even more complex. The bio-centric variable when considering industrial activities in nature explains both the water and biodiversity related issues variables under pollution and environmental challenges. Moreover, the biodiversity related issues variable is one of the explanatory variables for the bio-centric variable. This means that the relationship between the *DSP worldview* and the biodiversity related issues variable is modified. In addition, we see that the water related issues variable is one of the explanatory variables for the anthropocentric variable, thereby also weakening the correlation between the *NEP worldview* and the water related issues variable.

### 4.2. Muddling through conflicting values

The regression analyses reflect a mixed pattern, hinting at processes that might steer farmers onto trajectories characterised by either pro-environmental or human-centred values/attitudes. These processes most likely comprise contradictory factors as well as corresponding measures regarding environmental values and attitudes. Above, the potentially inherent contradiction of taking environmental protection measures and pursuing economic growth was underlined. This did not entail the well-being of nature as such, but assumed ‘nature was capable of recovering from human action’. The linkages between the *DSP worldview* and the biodiversity related issues variable, as well as between the *NEP worldview* and the anthropocentric variable and the production/economic variable, might cause the basic value to change or alter between the worldviews, as well as between different attitude variables representing pro-environmental or human-centred attitudes. Therefore, it is apparent that Chinese farmers are trying to balance diverging pressures of ‘the financial and the spiritual’.

This balancing or muddling through conflicting values has long historical roots in Chinese society, with on the one hand, the claimed environmental loftiness of Buddhism/Taoism [42, 43] or Confucianism [44], and on the other hand, the claimed mastery and exploitation of the environment conducted in daily practices [45–47]. Or a kind of middle position [48], claiming that the main focus of traditional Chinese thinking is how to maintain political unity and social harmony.

Processes of shaping and reshaping environmental values and attitudes take place in concrete settings that to a certain degree are contextual and formed in the interaction between driving forces at different spatial and temporal scales. Regarding the environment, especially in the eastern core of China because of relatively strong population pressure on existing resource, the rural areas have for centuries subsisted on the margin of their ecological niche, with over-exploitation of renewable resources (eco-system services) as a necessity for survival. This applies to the Yuqiao area, with the reservoir being man-made and with water transferred from Hebei province. Consequently, there are long-term historical traditions for how to handle challenges of physical constraints which gradually have changed the conditions for farming, developing an upper soil layer with little phosphorus retention capacity.

Farmers might be sympathetic to the environment, expressing pro-environmental values and attitudes, but they are also governed by their lack of opportunity to make the right choices and trade-offs between environment and their subsistence, guided by values and attitudes regarding how best to conduct farming. These values and attitudes are as indicated in the analyses also mixed. Set directly up against each other we find e.g. a stronger support for production output and income than an environmentally sound production and having healthy animals. Phosphorus is generally considered to be important for increasing farm output, and, consequently, values and attitudes linked to production are more important than water quality of Yuqiao, but not water for own consumption.

As underlined in the introduction, environmental *values* rarely explain environmental actions, and we have not found them to do so in Yuqiao either [9]. *Attitudes* may play a more important role intertwined with or alongside some of the background variables. For instance, the variables CPC membership, Production/economy motives of farming and partly Environment/health motives explain taking actions [9], and so represent channels between values and actions. This, again, supports strategies and efforts for improving farmers’ environmental knowledge as part of their farming competence; that is, developing environmental literacy.

## 5. Conclusions

The study confirms the usefulness of the NEP scale for analysing environmental values in different cultures, and the analysis shows a stronger support for a *NEP worldview* than *DSP worldview* among farmers in China. The segment of potential environmentalist farmers is thus higher than the segment of farmers advocating a human-centred exploitation of the environment.

Although females are more supportive of the *NEP worldview* than males, and the elders more supportive of the *DSP worldview*, these background variables are not consistent; that is, males are not more supportive of the DSP worldview and the young not more supportive of the NEP worldview.

The study shows that there is a grey zone, or fluidity between pro-environmental values and attitudes and human-centred values and attitudes. True, there are clear linkages between the *NEP worldview* and pro-environmental attitudes, but some linkages cross the dividing line between being pro-environmental and advocating a human-centred exploitation of nature. Over time, therefore, farmers’ values and attitudes may tilt from one trajectory to another.

This opens up scope for policy-making. Given the opportunity, farmers will adhere to environmentally sound practices. For instance, farmers will respond positively to the introduction of systems for collection of dung from husbandry and sewage. Taking into account the environmental conditions for farming in the area, we argue that the dominance of pro-environmental values and attitudes might also facilitate developing, learning, and introducing more environmentally sound farming. This should be part of a larger environmental-agronomic education programme for farmers, to enhance farming competence and prepare China for a modernised and sustainable farming for the future.

## Disclosure statement

No potential conflict of interest was reported by the authors.

## Funding

This work was supported by the Research Council of Norway for the SinoTropia project [project number 209687/E40].

## Notes

1. The ministry is also underlining that many actions have been taken to abate pollution and mitigate its effects; a fact that has its place in any overall judgements.

2. The literature on the NEP scale has established the technical term ‘item’ when referring to particular statements. We will use the more common term ‘statement’.

3. Tianjin city is near to Beijing. The two form the Beijing–Tianjin urban agglomeration. Yuqiao is part of the peri-urban section of this region.

4. The clay is halloysite; a type of clay that does not crack upon drying – thus being virtually impermeable.

5. About 68% of the farmers taking part in the survey reported having experienced water pollution; about 50% reported bad taste, smell and colour; and 19% reported having become sick from drinking water. Water pollution was considered as an important feature of agriculture by more than 60%.

6. For age: 30 and below: 21.5%; 31–40:17.7%; 41–50: 24.5%; 51–60: 22.2%; 60 and above: 14.1%.

## Annex 1: Background variables for the multivariate regression models

**Table:**

Variable	Values	Type
Gender	1: females (72.0%); 2: males (28.0%) (*N* = 496)	Nominal
Year of birth	Years; 1951–1952–1953, etc.	Numerical
Own education	1: No formal (7.3%); 2: Primary (18.9%); 3: Secondary (47.6%); 4: High school (20.3%); 5: College/university (5.9%) (*N* = 454)	Ordinal
Jobs outside farming	1: Only farming (33.8%); 2: farming and want to work outside (26.9%); 3: Working outside farming (39.3%) (*N* = 494)	Ordinal
Family income	Total income for all family members	Numerical
Social status village	1: Bottom (17.3%); 2: Middle-low (18.0%); 3: Middle-high (20.0%); 4: Close to top (16.4%); 5: Top (28.3%) (*N* = 434)	Ordinal
CPC membership	1: none (74.4%); 2: One or both parents (19.9%); 3: Husband or wife (3.9%); 4: husband or wife plus one/both parents (1.0%); 5; Husband/wife plus one/both parents (0.8%) (*N* = 508)	Ordinal
Village poverty rate	Percentage of respondent’s family in each village below 60% of median family income	Numerical

## Annex 2: Reliability statistics and factor analyses

**Table A**. The NEP scale.

**Table:**

*N* (valid) = 325 (183 cases excluded)		Reliability measures		Oblimin pattern matrix of Factor models		
	Mean score	Standard deviation	Item-total correlation	Factor Model-1	Factor Model-2	Factor Model-3
We are approaching the limit of the number of people the earth can support	3.29	1.514	.420	***.482***	.100	−.223
Humans have the right to modify the natural environment to suit their needs	3.25	1.435	.475	−.032	.191	***.608***
When humans interfere with nature it often produces disastrous consequences	3.54	1.477	.479	***.397***	.026	−*.345*
Human ingenuity will insure that we do NOT make the earth unliveable	3.44	1.722	.491	.052	***.425***	*.409*
Humans are severely abusing the environment	3.68	1.486	.481	***.440***	.053	−*.321*
The earth has plenty of natural resources if we just learn how to develop them	3.48	1.586	.580	−.222	***.417***	.178
Plants and animals have as much right as humans to exist	3.81	1.450	.635	***.594***	−.125	−.138
The balance of nature is strong enough to cope with the impacts of modern industrial nations	3.15	1.486	.459	.010	***.581***	.072
Despite our special abilities humans are still subject to the laws of nature	3.15	1.465	.486	.163	−***.401***	−.096
The so-called “ecological crisis” facing humankind has been greatly exaggerated	2.87	1.447	.398	.040	***.590***	−.020
The earth is like a spaceship with very limited room and resources	3.66	1.488	.591	***.639***	−.116	.010
Humans were meant to rule over the rest of nature	3.12	1.479	.476	−.023	***.700***	−.088
The balance of nature is very delicate and easily upset	3.74	1.443	.584	***.758***	−.135	.202
Humans will eventually learn enough about how nature works to be able to control it	3.44	1.476	.583	−.287	***.487***	−.024
If things continue on their present course, we will soon experience a major ecological catastrophe	3,74	1.419	.518	***.700***	−.053	.136
Eigenvalue				5.292	1.552	1.181
Percentage of variance				35.3	10.3	7.9

Extraction Method: Principal Axis Factoring, Rotation Method: Oblimin with Kaiser Normalization, Rotation converged in 7 iterations.

Note: Values in bold are the highest values above numeric value of .300, and italics above numeric value of .300 but not the highest.

**Table B**. The importance of environmental challenges for China.

**Table:**

*N* (valid) = 298 (210 cases excluded)		Reliability measures		Oblimin pattern matrix of Factor models		
	Mean score	Standard deviation	Item-total correlation	Factor Model-1	Factor Model-2	Factor Model-3
Air pollution	3.56	2.003	.541	***.530***	.021	.187
Heath waves	2.87	1.856	.612	***.454***	−.165	−128
Deforestation and desertification	3.33	2.115	.658	***.563***	−.297	−.226
Water scarcity and droughts	3.61	2.112	.632	***.722***	−.049	−.125
Polluted drinking water	3.56	2.119	.655	***.808***	.063	−.017
Waste water polluting rivers and lakes	3.64	2.068	.670	***.712***	.055	.192
Erosion of fertile soil	2.93	1.947	.750	***.429***	−*.384*	.067
Pollution of soil by toxic chemicals	3.34	2.054	.731	***.500***	−*.317*	.011
Loss of biodiversity	3.08	2.033	.705	.093	−***.730***	−.008
Poaching	2.96	2.057	.704	−.093	−***.918***	.020
Particular animal species becoming extinct	3.22	2.082	.738	.079	−***.760***	.034
Handling of waste from production and consumption	3.49	2.099	.693	.131	−***.526***	.274
Noise from transportation and industrial production	3.48	2.035	.587	.133	−.232	***.671***
Eigenvalue				6.850	1.006	.887
Percentage of variance				52.7	7.7	6.8

Extraction Method: Principal Axis Factoring, Rotation Method: Oblimin with Kaiser Normalization, Rotation converged in 11 iterations.

Note: Values in bold are the highest values above numeric value of .300, and italics above numeric value of .300 but not the highest.

**Table C**. Features of the environment to be taken into account when planning and implementing industrial activities in the natural environmental.

**Table:**

*N* (valid) = 392 (116 cases excluded)	Reliability measures			Oblimin pattern matrix of Factor models		
	Mean score	Standard deviation	Item-total correlation	Factor Model-1	Factor Model-2	Factor Model-3
The presence of endangered animal species	4.10	2.038	.686	.035	−***.866***	−.020
The presence of endangered plant species	4.31	1.945	.738	−.027	−***.770***	.187
The presence of a particular biotope and ecosystem, as a wetland	4.22	1.954	.679	***.478***	−*.365*	−.027
How untouched by humans the biotope is	3.92	1.989	.646	***.790***	−.021	−.017
The economic value of biotopes and species for human industrial production	4.07	1.960	.650	***.720***	.065	.141
The cultural and historical value of a landscape, biotopes and particular species for the human society	4.21	1.969	.603	−.004	−.013	***.756***
The scenery of a particular landscape and its economic value for tourism	4.10	1.945	.603	.061	−.042	***.646***
Eigenvalue				4.029	.762	.736
Percentage of variance				57.6	10.9	10.5

Extraction Method: Principal Axis Factoring, Rotation Method: Oblimin with Kaiser Normalization, Rotation converged in 8 iterations.

Note: Values in bold are the highest values above numeric value of .300, and italics above numeric value of .300 but not the highest.

**Table D**. Motives for practising farming.

**Table:**

N (valid) = 346(142 cases excluded)	Reliability measures			Oblimin pattern matrix of Factor models		
	Mean score	Standard deviation	Item-total correlation	Factor Model-1	Factor Model-2	Factor Model-3
Have a well-run field and tidy home/village	4.88	1.884	.530	***.663***	−.096	.040
Achieve high productivity and high output	5.06	1.724	.541	***.896***	.058	.074
Achieve high quality on the food products	5.07	1.690	.609	***.657***	−*.043*	−.111
Earn a high income as possible	5.05	1.725	.504	***.583***	.071	−.165
Produce food for own consumption	5.09	1.652	.545	.134	−.043	−***.505***
Have healthy animals	4.76	1.915	.575	−.034	.064	−***.836***
Have an environmental sound production	4.90	1.803	.624	.002	−.144	−***.638***
Learn and develop production techniques	4.91	1.796	.671	.289	−***.305***	−.255
Achieve model farmer status	3.77	2.224	.673	.084	−***.746***	−.054
Recognised by other farmers for good farming	3.69	2.254	.604	−.073	−***.981***	.060
Recognised by authorities for good farming	3.73	2.255	.601	−.065	−***.890***	.005
Hand over a well-run farm to next generation	4.55	2.029	.560	.117	−***.502***	−.095
Eigenvalue				5.343	1863	.851
Percentage of variance				44,5	15.5	7,1

Extraction Method: Principal Axis Factoring, Rotation Method: Oblimin with Kaiser Normalization, Rotation converged in 8 iterations.

Note: Values in bold are the highest values above numeric value of .300, and italics above numeric value of .300 but not the highest.
